# The problem of axonal injury in the brains of veterans with histories of blast exposure

**DOI:** 10.1186/s40478-014-0153-3

**Published:** 2014-11-25

**Authors:** Jiwon Ryu, Iren Horkayne-Szakaly, Leyan Xu, Olga Pletnikova, Francesco Leri, Charles Eberhart, Juan C Troncoso, Vassilis E Koliatsos

**Affiliations:** Department of Pathology, Division of Neuropathology, Johns Hopkins University School of Medicine, Baltimore, MD 21205 USA; Department of Neuropathology & Ophthalmic Pathology, Joint Pathology Center, Defense Health Agency, 606 Stephen Sitter Ave., Silver Spring, MD 20910 USA; Department of Neurology, Johns Hopkins University School of Medicine, Baltimore, MD 21205 USA; Department of Psychiatry and Behavioral Sciences, Johns Hopkins University School of Medicine, Baltimore, MD 21205 USA; Department of Psychology, University of Guelph, Guelph, ON N1G 2 W1 Canada

**Keywords:** Traumatic brain injury, Diffuse axonal injury, APP, Axon bulbs, Opiate, Microglia

## Abstract

**Introduction:**

Blast injury to brain, a hundred-year old problem with poorly characterized neuropathology, has resurfaced as health concern in recent deployments in Iraq and Afghanistan. To characterize the neuropathology of blast injury, we examined the brains of veterans for the presence of amyloid precursor protein (APP)-positive axonal swellings typical of diffuse axonal injury (DAI) and compared them to healthy controls as well as controls with opiate overdose, anoxic-ischemic encephalopathy, and non-blast TBI (falls and motor vehicle crashes).

**Results:**

In cases with blast history, we found APP (+) axonal abnormalities in several brain sites, especially the medial dorsal frontal white matter. In white matter, these abnormalities were featured primarily by clusters of axonal spheroids or varicosities in a honeycomb pattern with perivascular distribution. Axonal abnormalities colocalized with IBA1 (+) reactive microglia and had an appearance that was distinct from classical DAI encountered in TBI due to motor vehicle crashes. Opiate overdose cases also showed APP (+) axonal abnormalities, but the intensity of these lesions was lower compared to cases with blast histories and there was no clear association of such lesions with microglial activation.

**Conclusions:**

Our findings demonstrate that many cases with history of blast exposure are featured by APP (+) axonopathy that may be related to blast exposure, but an important role for opiate overdose, antemortem anoxia, and concurrent blunt TBI events in war theater or elsewhere cannot be discounted.

**Electronic supplementary material:**

The online version of this article (doi:10.1186/s40478-014-0153-3) contains supplementary material, which is available to authorized users.

## Introduction

Blast injury to brain, the signature injury of recent conflicts in Iraq (Operation Iraqi Freedom; OIF) and Afghanistan (Operation Enduring Freedom; OEF), is a hundred-year old problem that began with the introduction of trinitrotoluene in artillery shells in WWI [1]. Based on recent Department of Defense estimates [2], there are presently upwards of 250,000 OEF/OIF veterans with traumatic brain injury (TBI) history, many of them due to blast, and the importance of understanding the long-term sequelae of blast TBI cannot be overestimated. Blast TBI is complex and incorporates the direct effects of overpressure wave (primary injury), the gunshot-like effects of debris and shrapnel showering the head (secondary injury), the fall impact from translocation of the body by the overpressure wave (tertiary injury), as well as flash burns from the intense heat and asphyxiation or inhalation injuries [3]. Concurrent lesions to other organs leading to respiratory or heart failure or severe hemorrhage may contribute to brain hypoxia/ischemia. Factors that further complicate the long-term outcomes of blast injury are the complex dynamics of blast wave propagation, the mitigating or potentially enhancing effects of personal protective equipment [3], the number of blast exposure events [4,5], as well as the associated comorbidities of PTSD, chronic pain and substance abuse [6-8].

Despite the hundred-year long history of blast TBI and its significance for military medicine and beyond, its neuropathology has been very poorly characterized [9]. Part of the problem is lack of sufficient high-quality autopsy material, especially from long-term survivors of blast injuries. Recent reports from animal models of blast suggest the presence of axonal injury in select CNS tracts [10,11], but the occurrence or significance of axonal injury in the brains of human subjects with blast exposure is still a matter of debate [4,12].

Diffuse Axonal Injury (DAI) is a common event in human TBI, ranging from mild (concussive) to severe, including lethal, TBI [13-17]. DAI is a convention used for multifocal injury of long axons associated with impulse loading and encountered primarily in humans and other gyrencephalic animals [18]. DAI is featured by progressive axolemmal and axoskeletal alterations leading from axonal swellings (bulbs) to axotomy and the formation of retraction balls [19,20]. DAI was initially identified with silver degeneration methods [21]; however, because many neuronal proteins, including neurofilament proteins and amyloid precursor protein (APP) accumulate in damaged axons, immunohistochemistry (IHC) for these proteins has largely replaced silver methods in recent studies [14,19,22-24]. This paper directly documents the presence of APP (+) DAI in the brains of veterans with blast exposure history and raises important issues of interpretation and differential diagnosis.

## Materials and methods

### Subjects

Subjects were veterans with prior history of blast (*n* = 5; all males; ages 23-38, average 28; three of these subjects had died by opiate and/or alcohol overdose); controls with history of opiate overdose (*n* = 6; 2 males, 4 females; ages 18-48, average 25) or anoxic-ischemic encephalopathy (*n* = 6; 3 males, 3 females; ages 16-45, average 28); TBI controls with history of motor vehicle crashes (MVC) or falls (*n* = 5; all males; ages 18-51, average 31); and controls without history of TBI, opiate overdose or anoxic-ischemic encephalopathy (*n* = 7; 4 males, 3 females; ages 21-70, average 36) (Table 1). Brains were provided through the Armed Forces Institute of Pathology (AFIP) autopsy service or the Johns Hopkins Brain Resource Center (BRC). Blast cases included in this study did not have, as far as we know, contusional injuries. Essential demographic and clinical data as well as main neuropathological findings are summarized in Table 1. In addition, we used cases with Alzheimer’s disease and dementia pugilistica from the BRC collection as positive controls for phosphorylated tau IHC (see below).

**Table 1:** **Demographic**, **clinical and neuropathological signatures of subjects included in this study**

**Case**	**Age/Gender**	**Cause of death**	**Combat history**	**Blast/TBI history**	**Opiate overdose history**	**Anoxic-Ischemic brain changes**
1	38M	Multi-organ failure	Yes	Blast 2 mo PTD^1^	No	Purkinje Cell Degeneration
2	23M	GSW of head	Yes	Blast 11 mo PTD^2^	No	None
3	26M	Methadone overdose	Yes	Blast 1 yr PTD	Yes	Purkinje Cell Degeneration
				Concussion due to Assault 2 mo PTD^3^		
4	28M	Methadone overdose	Yes	Blast 4 yrs PTD^4^	Yes	CA1 Cell Degeneration
						Purkinje Cell Degeneration
5	25M	Alcohol and Opiate overdose	Yes	Blast PTD unknown	Yes	Purkinje Cell Degeneration
6	20M	Methadone overdose	No	No^5^	Yes	None
7	24M	Opiate overdose	No	No	Yes	CA1 Cell Degeneration
		Acute brain swelling				Purkinje Cell Degeneration
8	48F	Methadone overdose Acute hypoxic-ischemic encephalopathy <24 h duration	No	No	Yes	CA1 Necrosis
						Purkinje Cell Necrosis
						Cortical Necrosis
						Basal Ganglia Infarct
9	38F	Methadone and cocaine overdose Cerebral edema Early hypoxic-ischemic encephalopathy 12 h duration	No	No	Yes	CA1 Necrosis Neocortical and Entorhinal Necrosis
10	32F	Opiate overdose Cerebral edema Hypoxic-ischemic encephalopathy 48 h duration	No	No	Yes	CA1 Necrosis Neocortical Necrosis
11	18F	Opiate and antidepressant overdose	No	No	Yes	N/A
12	22F	Acute hypoxic-ischemic encephalopathy 27 d duration	No	No	No	CA1 Necrosis Purkinje and Deep Nuclei Cell Necrosis Cortical Necrosis Basal Ganglia Cell Necrosis
13	26M	Cardiac arrhythmia	No	No^6^	No	CA1 Cell Degeneration
		Acute hypoxic-ischemic encephalopathy				Purkinje Cell Degeneration
14	19M	Hypoxic-ischemic encephalopathy	Yes	Blast 19 d PTD^7^	No	CA1 Degeneration
						Purkinje Cell Degeneration
						Cortical cell death
						Basal ganglia cell death
15	16F	Hypoxic-ischemic encephalopathy (general anesthesia)	No	No	No	CA1 Necrosis
						Purkinje Cell Necrosis
						Neocortical Necrosis
						Basal ganglia Necrosis
16	45F	Hypoxic-ischemic encephalopathy Pneumonia Cardiac arrhythmia <24 h duration	No	No	No	CA1 Necrosis
						Purkinje Cell Necrosis
						Neocortical Necrosis
17	39M	Hypoxic-ischemic encephalopathy Asphyxia 4 d duration	No	No	No	CA1 Necrosis
						Purkinje Cell Necrosis
						Neocortical Necrosis
						Basal ganglia Necrosis
18	22M	Extensive traumatic brain injury	No	TBI due to MVA 12 d PTD^8^	No	Purkinje Cell Degeneration
		Brain hemorrhage				
19	21M	Intra cerebral hemorrhage	No	TBI due to Fall 4 d PTD^9^	No	CA1 Cell Degeneration
		Brain herniation				Purkinje Cell Degeneration
20	18M	Extensive TBI	No	TBI due to MVA 2 d PTD	No	N/A
21	42M	Extensive TBI	No	MVC 6 d PTD	No	N/A
22	51M	Extensive TBI	No	MVC 3 h PTD	No	N/A
23	21M	Sudden death Heavy lungs Negative toxicology	No	No	No	None
24	28M	Sudden death	No	No	No	None
		Normal autopsy				
		Negative toxicology				
25	21M	Sudden death	No	No	No	CA1 Cell Degeneration
		Stenosis of the coronary ostia				Purkinje Cell Degeneration
26	26F	Sudden death	No	No	No	CA1 and Cortical Cell Degeneration
		Mitral valve prolapse				Purkinje Cell Degeneration
27	70F	Sudden death	No	No	No	CA1 Cell Degeneration
		Cardiac arrhythmia				Purkinje Cell Degeneration
28	26M	Heart attack	No	No	No	None
29	48F	Heart attack	No	No	No	None

^1^IED; Other clinical problems: Burns 45% TBA; Blast lung; Lung inhalational injuries; Secondary bacterial and fungal infections, ^2^Multiple IED; Other clinical problems: PTSD, ^3^(IED) with noncontusional ejection 10 ft past explosion Other clinical problems: PTSD; Cognitive disorder secondary to TBI; Alcohol abuse, ^4^Other clinical problems: Postconcussive syndrome; PTSD; Depression; Adjustment disorder, ^5^Other clinical problems: Adjustment disorder with depression and anxiety; Migraine headaches, ^6^Other clinical problems: Multiple substance abuses, including inhalants and alcohol, ^7^IED; Other clinical problems: Chest injury; Ruptured spleen; Cardiac arrest, ^8^Related clinical findings: Multiple skull fractures; right frontal and temporal lobe contusions, ^9^Related clinical findings: Skull fracture; Left frontal/temporal contusions; subdural hematoma, PTD: Prior to death, CA1: Cornu ammonis 1 area in hippocampus, GSW: Gunshot wound, IED: Improvised explosive device, PTSD: Post-traumatic stress disorder, TBI: Traumatic brain injury, MVA: Motor vehicle accident, TBA: Total body area.

### Preparation of tissues, histology and IHC

Brains were immersion fixed in 10% formalin for two weeks at room temperature. Coronal tissue slabs were processed and embedded in paraffin and serial 10 μm-thick sections from standard blocks through frontal parasagittal cortex, anterior corpus callosum, hippocampus, thalamus, dorsolateral pons at the level of the superior cerebellar peduncle and cerebellar cortex were stained with hematoxylin and eosin, Cresyl violet, Masson’s trichrome, and processed for the IHC detection of: amyloid precursor protein (APP) (using a monoclonal antibody against the amino-terminus of APP [clone 22C11; MAB348 from Millipore, Temecula, CA] concentrated 1:200) as a marker of abnormal axons; glial fibrillary acidic protein (using a rabbit monoclonal antibody against the C-terminal of GFAP [clone EP672Y; 04-1031 from Millipore] concentrated 1:500) as marker of astrocytes; ionized calcium- binding adaptor molecule 1 antibody (IBA1; a rabbit polyclonal antibody [CP290A from Biocare Medical LLC, Concord, CA] concentrated 1:300) as microglial marker; and mixed phosphorylated neurofilament epitopes (a mouse monoclonal antibody against phosphorylated neurofilament H and, to a limited extent, neurofilament M [SMI-310R; Covance, Emeryville, CA] concentrated 1:200) as generic axonal marker.

Sections through the frontal cortex and hippocampus from all blast cases were also processed for phosphorylated tau IHC with antibodies AT8, PHF1, and CP13 and the conformational tau antibody MC1. AT8 is directed against tau pS202 (MN1020; Thermo Scientific Inc., Rockford, IL); PHF1 is directed against tau pS396 and pS404; CP13 is directed against tau pS202 and pT205; and the conformational antibody MC1 is directed against the third microtubule binding domain of tau (amino acids 313 to 322) and recognizes the conformation formed by N-terminus amino acid 7 to 9 and amino acids 313 to 322 in neurofibrillary tangles, but not fetal Alz-50-reactive clone 1. Antibodies PHF1, CP13 and MC1 were provided by Dr. Peter Davies, Albert Einstein College of Medicine, Bronx, NY. All antibodies were used in 1:200 concentration. Normal IgG from the species of origin of the primary antibodies was used as negative control.

Immunoperoxidase IHC was performed essentially as described in Hedreen and Koliatsos (1994) [25] with some modifications. Paraffin sections were deparaffinized and rehydrated by incubation at 60°C for 30 minutes followed by sequential treatments with xylene, 100% and 95% ethanol. Endogenous peroxidase activity was blocked with hydrogen peroxide. Three percent (w/v) normal IgG in TBS including 0.1% Tween 20 solution was used for blocking and antibody incubation. The appropriate biotinylated goat anti-primary antibody IgGs in concentrations 1:250 to 1:400 were used as secondary antibodies. A standard avidin-biotin peroxidase reaction (PK-6100, Vector Lab. Inc., Burlingame, CA) followed by diaminobenzidine (DAB) incubation were used to visualize immunoreactive epitopes. For double IHC of IBA1 or GFAP with APP, APP immunoreactivity was detected first as stated previously and IBA1 or GFAP immunoreactivity was detected by ImmPRESS-AP Anti-rabbit IgG (MP-5401) or a Vector blue alkaline phosphatase substrate kit (SK-5300), both from Vector Lab. In some cases, we also performed silver staining with a variation of the Gallyas procedure using NeuroSilver™ Kit II (PK301A) from FD NeuroTechnologies (Ellicott City, MD).

### Counts of APP (+) axonal swellings in cases with blast histories or methadone overdose

Because the vast majority of cases with blast histories had died of opiate overdose, the relationship between blast exposure and APP (+) axonal pathology was further studied by comparing between cases with blast histories and cases with opiate overdose prior to death. Axonal swellings were visualized and counted on APP-immunostained sections of the superior frontal and cingulate gyri. The parasagittal white matter in these areas was outlined on one representative coronal section per case of subjects exposed to blast (*n* = 4) and subjects who died from opiate overdose (*n* = 4). The magnitude of APP (+) axonal pathology was measured with the areal fraction fractionator probe [26] using the Stereo Investigator software (MicrobrightField Inc., Williston, VT). The fractional area of APP (+) axonal swellings was generated per case and then combined in the blast and opiate overdose groups that were further compared using a student’s t-test.

## Results

### APP (+) axonal abnormalities in subjects with blast history

**Figure 1:**
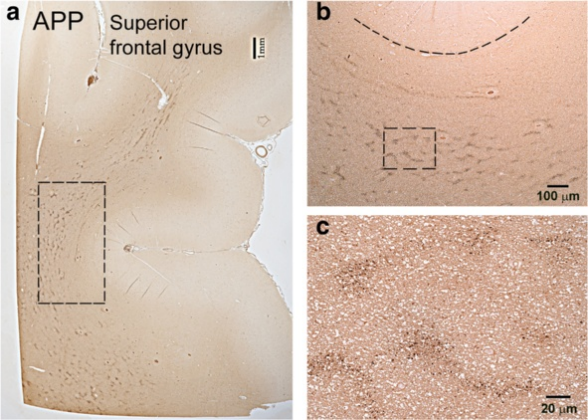
**The typical honeycomb pattern of APP** **(+)** **staining in the superior frontal gyrus of an index case with a history of blast injury (Case 3, Table** **1** **). (b)** and **(c)** are enlargements of the blocked areas in **(a)** and **(b)**, respectively. This case had blast exposure 1 year before death and a concussion from assault 2 months prior to death. Note the groups of tens-hundreds of axonal spheroids in the deep white matter of frontal lobe.

In all cases with history of blast exposure except one (Case 2), the most consistent finding was APP-immunoreactive axonal varicosities or spheroids in the medial (parasagittal) frontal white matter, primarily in the superior and middle frontal and cingulate gyri (Figure 1). These lesions clustered in circular or semi-circular formations at short distances (50-200 μm) from blood vessels and frequently coalesced in irregularly shaped lattices forming honeycomb patterns (Figure 1a-b). Similar patterns were often seen in the white matter of the anterior and middle temporal lobe. No parietal lobe tissues were available for review. The one case with blast history that did not show APP (+) axonal abnormalities in medial frontal white matter or elsewhere in the brain (Case 2) had a history of exposure to multiple IEDs and died from gunshot wound to the head (Table 1).

**Figure 2:**
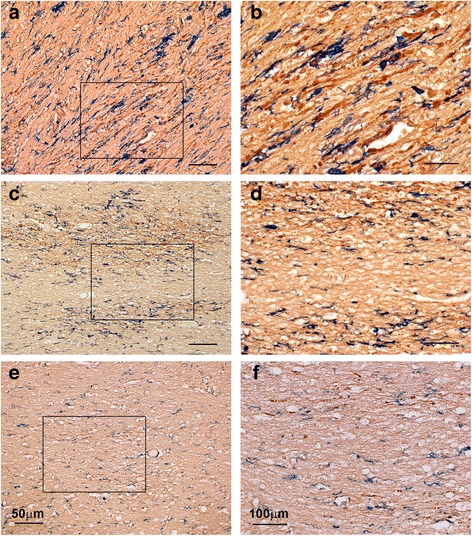
**Sections immunostained for concomitant localization of APP and IBA1 epitopes in representative cases of MVC (Case 21; a-b), blast (Case 1; c-d), and opiate overdose (Case 7; e-f).** APP (brown) and IBA1 (blue) was detected sequentially by DAB and Vector blue, respectively, as described in Methods. Note that, in MVC **(a-b)** and blast **(c-d)** APP (+) swellings and bulbs colocalise with deramified or phagocytic microglia, whereas in opiate overdose **(e-f)** they do not. Panels b, d, and f are magnifications of framed areas in a,c, and e, respectively. Size bars: 50 **(a, c and e)** and 100 **(b, d, and f)** μm.

In some cases with blast histories, groups of abnormally swollen APP (+) axons and axon bulbs were also encountered in the corpus callosum (Additional file 1: Figure S1 for Case 3 but also Figure 2 for Case 1), the superior cerebellar peduncle, and cerebellum. In the cerebellum, axonal swellings were often seen in the deep white matter next to deep nuclei and also in the form of classical retraction balls in the granule cell layer next to Purkinje cell bodies. These retraction balls were eosinophilic, argyrophilic, and APP-immunoreactive, and were also filled with phosphorylated neurofilaments (Additional file 2: Figure S2 for Case 3). One of these cases, e.g. the one illustrated in Additional file 1: Figure S1 and Additional file 2: Figure S2, had suffered traumatic events other than blast relatively near the time of death. APP (+) axonal swellings were occasionally encountered also in internal capsule, striatopallidal pencils, and thalamic striae.

**Figure 3:**
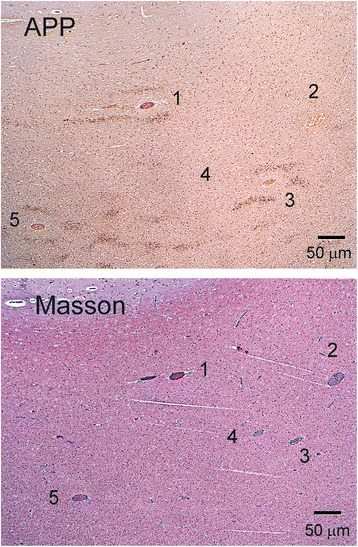
**Perivascular distribution of APP-immunoreactive axon abnormalities demonstrated here on adjacent sections, one of which (top) was processed for APP immunohistochemistry and the other (bottom) with Masson trichrome stain (images taken from Case 3).** Numbers indicate corresponding blood vessels. Note that axonal abnormalities encircle aniline blue (+) thick-walled vessels representing arterioles (#1, 3, 5), but not thin-walled vessels that are venules (#2, 4). (4) is not evident on top section and only its position was marked as such. Size bars: 50 μm.

The comparison of sections immunostained with APP and adjacent sections stained with Masson’s trichrome in regions with honeycomb pattern of axonal lesions shows that APP (+) perivascular abnormalities are associated with arterioles, but not venules (Figure 3).

### APP (+) axonal abnormalities in subjects with history of opiate overdose, anoxic-ischemic encephalopathy and non-blast TBI

**Figure 4:**
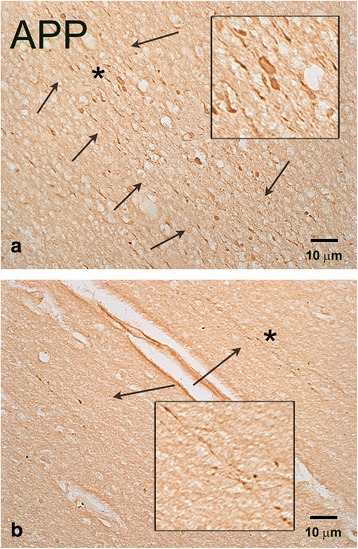
**APP (+) axonal abnormalities in the frontal lobe of a representative patient without history of blast who died as a result of opiate overdose (Case 9, Table** **1** **).** Although there are occasional clusters of axonal swellings (arrows, **a**), most cases of observed abnormalities are less conspicuous (arrows, **b**). Insets represent magnifications of areas marked with an asterisk. Size bars: 10 μm.

**Figure 5:**
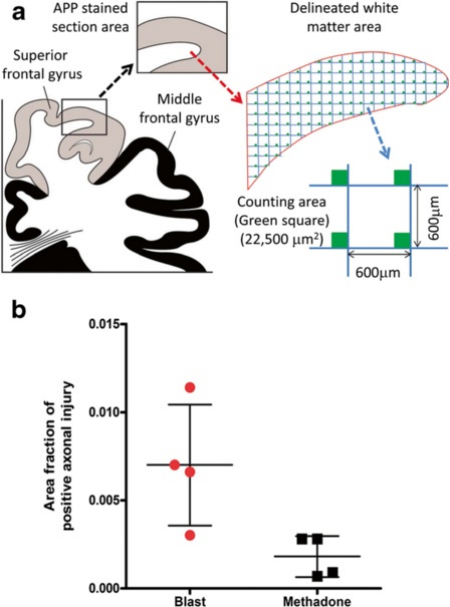
**Stereological areal fraction analysis of APP-positive axonal injury signal in blast (n = 4) and methadone (n = 4) cases.** Coronal sections through the frontal lobe were stained by APP N-terminal antibody (see Materials and Methods). Axon injury signal was counted **(a)** and analyzed by two-tail student’s t-test **(b)**. Difference between blast and methadone cases was significant at *P value 0.0195*.

Because 3 out of 4 blast cases with APP (+) axonal swellings had died of opiate overdose (Table 1), 6 additional cases with opiate overdose without blast exposure were analyzed as controls. In 5 out of 6 subjects, we observed a few APP (+) axonal abnormalities in the medial frontal white matter, corpus callosum and the internal capsule (Figure 4), but these pathologies were significantly less pronounced compared to the ones in blast injury cases. In one case of an 18-year old female (Case 11) who died from mixed overdose with antidepressants and opiates, we found extensive axonal abnormalities in patches throughout the white matter of frontal cortex. Based on the mixed nature of intoxication that may be associated with regulatory APP events [27] this brain was not included in the opiate overdose group for further stereological analysis, but was illustrated in supplementary figures (Additional file 3: Figure S3). When we analyzed the density of APP (+) axonal abnormalities in frontal cortex with stereology, there were significant differences between the blast (n = 4) and methadone (n = 4) groups (P = 0.0195; Figure 5). The honeycomb pattern of axonal injury with perivascular distribution (Figure 1) was not evident in methadone overdose cases.

In cases with anoxic-ischemic encephalopathy, APP staining of axons was extremely variable. Two cases of young adults showed rare APP (+) fiber abnormalities in medial frontal white matter and temporal lobe or brain stem (Cases 12 and 13), whereas four other cases were negative for APP signal in frontal lobe. The brain of a 16-year old patient (Case 15) who died in the course of anesthesia and had brain edema with herniation showed dense APP (+) fibers in brain stem, cerebellar white matter, medial temporal lobe (angular bundle), and internal capsule/striatopallidal pencils, but no staining in medial frontal white matter. We also reviewed two cases with exposure to methadone overdose at ages 2 and 3 (not included in Table 1). Both had medial cortical necrosis and displayed APP (+) long fiber swellings coursing vertical to the white-gray matter border and along the longitudinal axis of the gyrus that was different from the abnormalities seen in blast and opiate overdose material.

Cases 18 and 19 with cerebral contusions demonstrated axonal pathology primarily at the border of the contusion (Additional file 4: Figure S4); none had APP (+) abnormalities in the medial frontal white matter. Cases 20 and 21 with histories of MVC showed large numbers of abnormal axons with swellings and axon bulbs cursing in multiple areas, especially in the medial frontal white matter and anterior corpus callosum, without clear association with blood vessels (Additional file 5: Figure S5 for Case 20). No evident APP (+) axonal abnormalities were seen in controls with negative neuropsychiatric histories.

**Figure 6:**
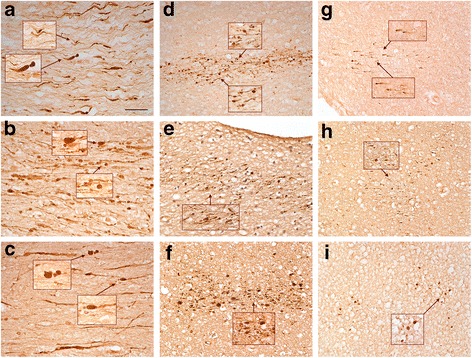
**Microscopic features of APP (+) axon abnormalities in motor vehicle crashes (a-c), blast (d-f) and opiate overdose (g-i) in typical cases. a** is taken from Case 20, **b** and **c** from Case 21, **d** from Case 4, **e** from Case 5, **f** from Case 3, **g** from Case 9, **h** from Case 7 and **i** from Case 6. Insets represent enlargements of indicated areas. Note differences in size of axons among the three types of injury and also the prominence of undulations and axon bulbs in cases of motor vehicle crash **(a-c)**. Portions of the insets are further magnified in Figure 7. Size bars: 50 μm.

**Figure 7:**
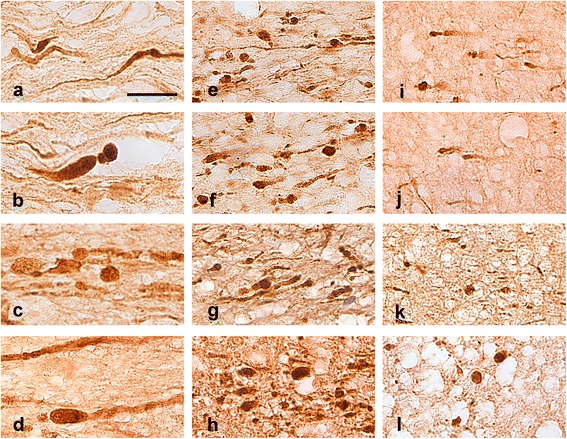
**Further magnification of areas indicated with arrows in Figure** **6** **.** Arrangement of panels is exactly as in Figure 6. These images illustrate in greater detail the anatomical differences of injured axons among classical traumatic DAI (**a** and **b** from Case 20, **c** and **d** from Case 21), blast (**e** and **f** from case 4, **g** from Case 5, **h** from Case 3), and opiate overdose (**i** and **j** from Case 9, **k** from Case 7, **l** from Case 6). Size bars: 20 μm.

As shown in Figures 6 and 7, the microscopic appearance of axonal abnormalities is different among cases with histories of motor vehicle crash (the classical scenario causing DAI) versus blast versus opiate overdose. In coronal sections like the ones used in this study, axonal abnormalities in cases of motor vehicle crash are thick with prominent undulations and classical axon bulbs; in the case of opiate overdose, axonal abnormalities are thin and straight, with multiple varicosities in the course of a single axon; in blast cases, axonal abnormalities fall in-between classical DAI and opiate overdose cases and feature prominent varicosities and spheroids (Figure 6 and, in further magnification, Figure 7).

### Neuroinflammatory responses and other neuropathologies

**Figure 8:**
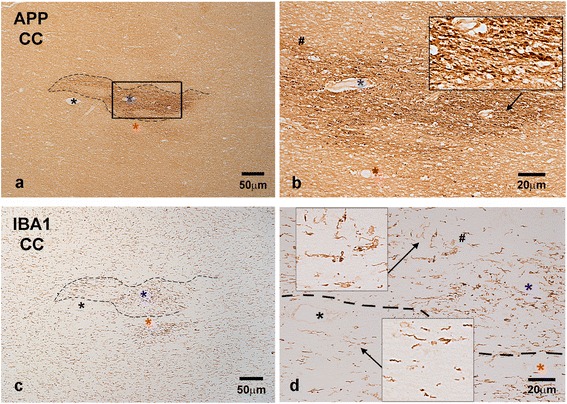
**Adjacent sections through the anterior corpus callosum of a typical blast case (Case 1; Table** **1** **) immunostained with an axonal injury marker (APP; a-b) and microglial marker (IBA1; c-d). b** is enlargement of bracketed area in a and shows, in greater detail, abnormally swollen axons traveling in various directions. **d** is an enlargement of bracketed are in **c** taken through the border between healthy tissue and a patch of axonopathy. Insets in d are magnifications of microglial profiles indicated with arrows. Note the correspondence of axonopathy **(a)** with neuroinflammation **(c)** in the same patch of tissue and the transition between ramified and deramified/round microglia in the border between reactive and non-reactive tissue in **(d)**. In Figure 2e-f, note that microglia has resting cytology and there are no appreciable differences between areas with APP (+) axonopathy and areas without. Size bars: 50 **(a and c)** and 20 **(b and d)** μm.

To explore the regional association of axonal abnormalities with neuroinflammatory responses in the brains of blast cases, we performed IHC for the microglial marker IBA1 or the astrocytic marker GFAP and compared such labeling with immunolabeling for APP on same or adjacent sections. Figure 8 shows evidence of colocalization of IBA1 (+) deramified/hypertrophic microglia with patches of abnormal APP (+) axons in the corpus callosum of an index case with blast history (Case 1). The best evidence of colocalization of APP (+) axonal abnormalities with activated microglia in blast cases is shown in double-labeling preparations as the one illustrated in Figure 2c-d (Case 1). A similar colocalization of hypertrophic, phagocytic microglia with APP (+) axonal abnormalities is seen in DAI associated with MVC (Figure 2a-b; Case 21). On the other hand, there is no evidence of microglial activation next to abnormal axons in cases of opiate overdose (Figure 2e-f; Case 7). In contrast to the colocalization of APP (+) axonal abnormalities with activated microglia in blast and MVC cases, there was no clear evidence for colocalization of such abnormalities with GFAP (+) reactive astrocytes.

Gross measures of hypoxia-ischemia were variable in our blast material; one out of five cases showed hippocampal CA1 degeneration, 4 cases showed apparentreduction in numbers of Purkinje cells in cerebellar cortex and none showed cortical necrosis. In comparison, 4 out of 6 cases of methadone overdose showed CA1 degeneration or necrosis, 2 cases showed Purkinje cell degeneration or necrosis and 3 cases showed cortical necrosis. On the other hand, all 6 cases of hypoxic-ischemic encephalopathy showed CA1 and Purkinje cell degeneration or necrosis, 5 cases showed cortical necrosis and 4 cases showed basal ganglia necrosis (Table 1). The severity of anoxic-ischemic changes in these three groups of cases appears to bear no association with the severity of axonal abnormalities in the medial frontal white matter.

To explore the possibility that blast exposure is associated with tauopathy as suggested in a recent study [28], we processed sections of the frontal and temporal lobes of 5 blast cases with IHC for distinct phosphorylated and conformational tau epitopes using antibodies AT8, CP13, PHF1 and MC1. We did not detect a positive signal in any of these blast cases (Additional file 6: Figure S6), including Case 4 that survived 4 years after blast, despite the fact that all antibodies reacted with neurofibrillary tangles in control AD cases (data not shown) and all antibodies except MC1 stained tangles in an established case of dementia pugilistica (Additional file 6: Figure S6a-f).

## Discussion

### Relevance of findings and comparison with other lines of research

Our findings show that brains of subjects with a history of blast exposure are featured by widespread APP (+) axonal abnormalities. In the cortical white matter, these abnormalities appear primarily as clusters of small axonal varicosities or spheroids. This form of DAI differs from classical DAI due to MVC or axonal degeneration associated with contusions. Although most of the brains with blast histories examined here belong to subjects who died from opiate overdose, the pathological patterns described here differ from axonal lesions in subjects that died from overdose without history of blast exposure. Differences are based on density, their degree of association with neuroinflammation, and also some microscopic features of axonal abnormalities. It is possible that prior blast or blunt TBI exposure and particular antemortem circumstances, such as opiate overdose and/or anoxia, may act in synergy to produce the honeycomb pattern of APP staining seen in Figure 1. The focus of this study was APP (+) axonopathy without any claims as to whether this is a primary or secondary outcome of blast. Although traumatic axonopathy has been confirmed by several groups working on animal models of blast TBI [10,11,29], some investigators place emphasis on microvascular pathology [30,31].

To our knowledge, this is the first direct evidence of DAI in the brains of veterans with prior blast exposure. Indirect evidence consistent with the presence of DAI comes from diffusion tensor imaging (DTI) studies on soldiers with blast histories. Mac Donald et al have reported abnormal DTI signal in the middle cerebellar peduncle, cingulum, and orbitofrontal white matter of U.S. military personnel within 90 days of injury [4], but only middle cerebellar peduncle abnormalities were replicated in a follow-up report focusing on pure primary blast injury [32]. In another DTI study, Jorge et al reported on regions of reduced low fractional anisotropy in the brains of OIF/OEF veterans with mild TBI. These lesions had a predilection for the corpus callosum and, less so, the cerebellum, and could be caused by axonal injury [12]. Another DTI study in veterans of OIF/OEF with histories of mild TBI showed similar patterns [33]. Yet other investigators failed to find DTI-based abnormalities in veterans with mild-to moderate blast TBI [34].

Anatomical regions highlighted in DTI studies partially overlap with the regional distribution of DAI lesions reported here. Our findings may show a greater involvement of the hemispheres than the cerebellum, but our limited sampling does not allow definitive conclusions. Furthermore, it is unclear how the several mm-diameter “potholes” of limited fractional anisotropy described by Jorge et al [12] might correspond to the much smaller honeycomb islands identified in the present study. Blast injury simulation modeling reveals maximal shearing stress in a few brain sites including orbitofrontal, brain stem and frontal parasagittal regions [35] and predicts, in part, neuropathological patterns observed here. The selective vulnerability of certain long CNS tracts to primary blast is supported by recent animal studies demonstrating traumatic axonal injury in the corpus callosum, corticospinal, cerebellar, and lemniscal pathways [10,11]. The involvement of these tracts may be related to the distribution and intensity of shearing stress and other types of mechanical distortion in the brain, although such biomechanical factors may operate differently in the human versus rodent CNS [35]. The peri-arterioral distribution of axonal pathology in our blast cases may be due to similar factors, for example shearing stress of axons may be especially intense in the white matter enveloping the mechanically stiff arterial environment.

### TBI-associated DAI versus opiate-overdose axonopathy

The majority of blast cases with APP (+) axonal swellings had died of opiate overdose. Brains of patients who died of opiate overdose without history of blast exposure did show evidence of axonopathy, but some features of blast-associated axonal injury set it apart from the injury associated with opiate overdose. First, the severity of APP (+) axonopathy is greater in blast cases compared to cases with opiate overdose without blast history. Second, there is close relationship of APP (+) axonal abnormalities with activated microglia in blast cases, even 4 years after the index event; this finding suggests the presence of neuroinflammation in regions with abnormal axons that takes days to evolve and may not be consistent with agonal events related to overdose as sufficient causes of blast-related axonopathy. Third, there are some microscopic differences between abnormal axons in cases of opiate overdose and cases with history of blast: the former present primarily as beaded, relatively thin axons, whereas the latter usually present as larger varicosities or spheroids. According to some studies, granular appearance of axons may represent advanced axonal degeneration with axon fragments persisting months or years after blast, in tandem with enduring neuroinflammation [36-38]. The longevity of axons or their fragments after lesions is not a rare phenomenon in neurobiology of disease [39] and some of the molecular mechanisms have been revealed in Wld mice [40,41]. Fourth, axonal abnormalities related to opiate overdose do not show the prominent peri-arteriolar honeycomb distribution of blast-associated axonal lesions.

Opiate overdose may contribute to the axonal abnormalities observed in blast cases. For example, primary blast injury may have caused endangerment of the axons or regional neuroinflammation that was further complicated by chronic use of opiates or by protracted agonal events associated with opiate overdose and culminated in the observed honeycomb pattern. In this scenario, honeycomb varicosities or spheroids may represent “fresh” low-grade axonopathy [42] in primed axons or in regions primed by chronic neuroinflammation, both from the original blast. Non-primary blast injury may also have contributed to the axonal abnormalities observed here, including tertiary injuries from concussions and quaternary thermal injuries. Traumatic events such as blunt concussions suffered outside the combat theater in the weeks prior to death may have also added to this pattern.

The association between opiate overdose and axonopathy has been reported before [43-45] and was largely ascribed to hypoxia. In our case series, brains with classical anoxic-ischemic encephalopathy had negligible axonal injury, a pattern consistent with previous studies [46]. Furthermore, rodents dying of methadone overdose do not show APP (+) axonal swellings (Leri, Koliatsos and Ryu, unpublished observations), despite the fact that traumatic axonal injury can be induced in a variety of rodent TBI models [11,47-51]. Of course, the absence of axonal injury in severe anoxic-ischemic encephalopathy does not preclude the presence of axonopathy in the case of prolonged milder hypoxia. At the same time, the absence of axonopathy in rodent models of opiate overdose does not preclude the occurrence of such in the course of opiate overdose in humans. Beading of axons in the absence of bulbs seen in opiate and other overdose cases may be associated with nodal or paranodal events, including swelling of mitochondria, as shown in experimental anoxic nerve injury [52]. The role of opiate overdose and/or anoxia in the evolution of honeycomb axonopathy in blast cases is also suggested by Case 2 that, although clearly exposed to blast, did not show APP (+) axonopathy. This patient died from self-inflicted gunshot wound to the head that we assume caused instant death, a condition incompatible with prolonged agonal state and brain anoxia. The presence of honeycomb axonopathy in Case 1 that had died of multi-organ failure, but not opiate overdose, is also compatible with a role of anoxia in the establishment of such lesions.

We propose that blast exposure and agonal hypoxia (primarily caused by opiate overdose in our series) should be viewed as interactive or synergistic factors in the genesis of the honeycomb axonopathy observed here. It is difficult to say whether the spherical lesions represent residues of progressively degenerating axons from the original blast event or, rather, fresh low-grade lesions caused by prolonged hypoxia in axons primed at the time of the blast. Such questions address the broader problem of the cumulative effect of multiple injurious impacts to the brain and, to some extent, can be modeled and studied in animals.

### Blast history and tauopathy

Two recent reports [28,53] have demonstrated the presence of tauopathy in the brains of five veterans with prior blast exposure and one of these studies [28] modeled this finding on single-blast exposure of mice featuring free head movement. The same study [28] reported axonal and microglial pathology next to tau deposits that are not directly comparable to our findings. Using an identical panel of phosphorylated tau antibodies as Goldstein et al [28], we were unable to confirm tau hyperphosphorylation in any of our 5 blast subjects, including a case that had survived 4 years after blast. Neuropsychiatric symptoms in our subjects were broadly similar to these of the other reports and involved a combination of PTSD, depression and cognitive difficulties. The varied findings may be due to a number of reasons including a limited number of sampling sites in our material, differences in tissue processing such as length of fixation, and differences in average time interval between blast events and death that was somewhat longer in the case series of Goldstein et al [28]. At the very least, the issue of tauopathy after military blast history is very complex and findings from one case or case series cannot be readily generalized. As in the case of other comparisons made here, there is great need for several types of controls, including adequate sampling of young or middle-aged subjects without blast or TBI history.

### Clinical considerations and problems in establishing cause and effect

Although it is risky to draw clinical conclusions based on pathological autopsy material, the strong frontal and paralimbic patterns of axonal injury reported here may shed light into the problem of chronic executive and attentional difficulties experienced by chronic survivors of blast TBI [54]. On another clinical subject, the non-intentional overdose by methadone, used as a popular analgesic by veterans, is a very important problem in retired military personnel [55,56] and it is possible that it may exacerbate pre-existing axonal injuries. This important issue should be clarified with further research.

Naturally, any study based on brain autopsy material from the complex cohort of OIF/OEF veterans should take into account a life style often featured by repeat combat and non-combat TBI and substance abuse/overdose. As highlighted in the previous sections of Discussion, these events may act in synergy to cause axonopathy. Time parameters are also important. Acute and chronic DAI have different microscopic appearances [36,38] and, at the same time, survival time also reflects TBI severity: Lethal TBI by definition involves brief survival and causes severe acute axonopathy featured by prolific axonal swellings with bulbs; mild TBI is not a cause of death per se and is naturally associated with fewer axonal lesions that fade over time or persist as granular residues. Thus, survival time may play an important role in the differences seen between DAI due to MVC and DAI in subjects with blast histories (Figures 6 and 7).

Our findings provide the groundwork for a fresh look into the problem of blast injury to brain, and stress the fact that DAI may be shared between blast and other types of acceleration injury to brain, although pathological details appear to be different. Importantly, our study underlines the complexity of cases with blast history that come to autopsy and the need to engage several different types of controls, while recognizing the possibility of interacting pathogenic factors. Simple blast cases featuring solely primary blast events without secondary, tertiary etc components and not associated with other combat or non-combat TBI or substance abuse comorbidities or antemortem hypoxia may lend themselves to more straightforward clinico-pathological analyses, but such cases are probably a small fraction of the TBI problem in the OIF/OEF veteran cohort and are extremely rarely represented in autopsy samples. While one may be tempted to establish clear cause-and-effect relationships based on simpler cases, it may be the cumulative impact of multiple pathogenic factors and morbidities that determines TBI-related outcomes in veterans of recent wars.

## Conclusions

Brains of subjects with history of blast exposure show a honeycomb pattern of APP (+) axonopathy, detected here in the medial dorsal frontal white matter. Most of these subjects had died of opiate overdose. Axonal abnormalities in blast subjects are affiliated with reactive microgliosis, a pattern that distinguishes them from control cases of subjects who died of opiate overdose but had no blast history. Blast-associated axonopathy is also denser compared to that seen in opiate subjects and appears to have some distinct microscopic features. It is possible that blast and concurrent blunt TBI exposure as well as particular antemortem circumstances, such as opiate overdose and/or anoxia, may act in synergy to produce the pattern of APP axonopathy observed here. Our study establishes the background for future work that should help clarify the previous mechanisms and provide more definitive insights into the hundred-year old problem of blast neurotrauma.

## Additional files

Additional file 1: Figure S1. APP (+) axonopathy in the corpus callosum of an index case with history of blast injury (Case 3, Table 1). Enlarged, fusiform axons and axonal bulbs are indicated with arrows in (a). (b) represents an enlargement of the blocked area in (a). It is unclear whether these patches of classical DAI are associated with blast or, rather, the concussion suffered as a result of assault 2 months prior to death. Additional file 2: Figure S2. Neuropathological findings in the cerebellum of an index case of blast injury (Case 3, Table 1) based on staining with hematoxylin-eosin (a), Gallyas silver (b), and phosphorylated neurofilament (c) or APP (d) immunohistochemistry. Note enlarged eosinophilic (a) or argyrophilic (b) or phosphorylated neurofilament- and APP-immunoreactive axon bulbs (c and d) in the Purkinje or granule cell layer next to the cell bodies of Purkinje cells (arrows). APP immunohistochemistry is especially sensitive in detecting these axonal abnormalities. Insets represent magnifications of areas indicated with asterisks. As in the case of classical axonal swellings with bulbs in Additional file 1: Figure S1, it is unclear whether these profiles are associated with blast or the concussion suffered from assault 2 months before death. GL, glomerular cell layer; ML, molecular layer; PCL, Purkinje cell layer. Size bars: 5 μm (a) and 20 μm (b, c and d). Additional file 3: Figure S3. This case of mixed drug overdose (Case 11 in Table 1) , including opiates, is associated with severe axonal injury that serves to illustrate better the type of axonopathy that occurs in drug intoxication. Although the pattern is qualitatively similar to that in opiate overdose, the intensity of labeling is unusually high. Note the straight thin axons with periodic round swellings (arrows in b). Size bars: 50 μm. Additional file 4: Figure S4. Patterns of APP (+) axonal pathology in brain contusions. Case illustrated here (Case 19, Table 1) had hemorrhagic contusion in ventromedial frontal lobe (a, arrows). Note the presence of multiple APP (+) dilated, short axon stumps (circles) in the periphery of hemorrhage (area indicated with an asterisk in a). Size bars: 500 (a) and 20 (b) μm. Additional file 5: Figure S5. APP (+) axonal pathology in cases of motor vehicle crashes is especially dense in medial dorsal frontal lobe (a, arrows). Images here correspond to case 20 (Table 1). Axonal abnormalities do not display perivascular patterns as in the case of blast injuries (b; this is an enlargement of framed area in a). Size bars: 1 mm (a) and 25 μm (b). Additional file 6: Figure S6. Phosphorylated tau (AT8, CP13 and PHF1) staining of three blast injury cases (g, h and i) compared with an established dementia pugilistica case used here as positive control (a to f). Although antibodies AT8, CP13 and PHF1 react intensely with phosphorylated tau epitopes in the of the dementia pugilistica case (a-c; d-f are magnifications of framed areas in a-c, respectively), no immunoreactivity is seen in the hippocampus of Case 5 with history of blast exposure (g-i). Sections through the temporal lobe of other blast cases were similarly negative and MC1 antibody did not stain brain tissues with a history of dementia pugilistica or blast (data not shown). Size bars: 2 mm (a, b, c, g, h, and i) and 50 μm (d, e, and f).

## Abbreviations

AD: Alzheimer’s disease
AFIP: Armed Forces Institute of Pathology
APP: Amyloid precursor protein
BRC: Johns Hopkins Brain Resource Center
CNS: central nervous system
DAB: diaminobenzidine
DAI: Diffuse axonal injury
DTI: Diffusion tensor imaging
GFAP: Glial fibrillary acidic protein
IBA1: Ionized calcium-binding adapter molecule 1
IHC: Immunohistochemistry
MVC: motor vehicle crash
OEF: Operation Enduring Freedom
OIF: Operation Iraqi Freedom
PTSD: post traumatic stress disorder
TBI: Traumatic brain injury
