# Supplementary figures and images for: The problem of axonal injury in the brains of veterans with histories of blast exposure
Source: Acta Neuropathol Commun. 2014 Nov 25;2:153. doi: 10.1186/s40478-014-0153-3 (PMC4260204; doi:10.1186/s40478-014-0153-3)

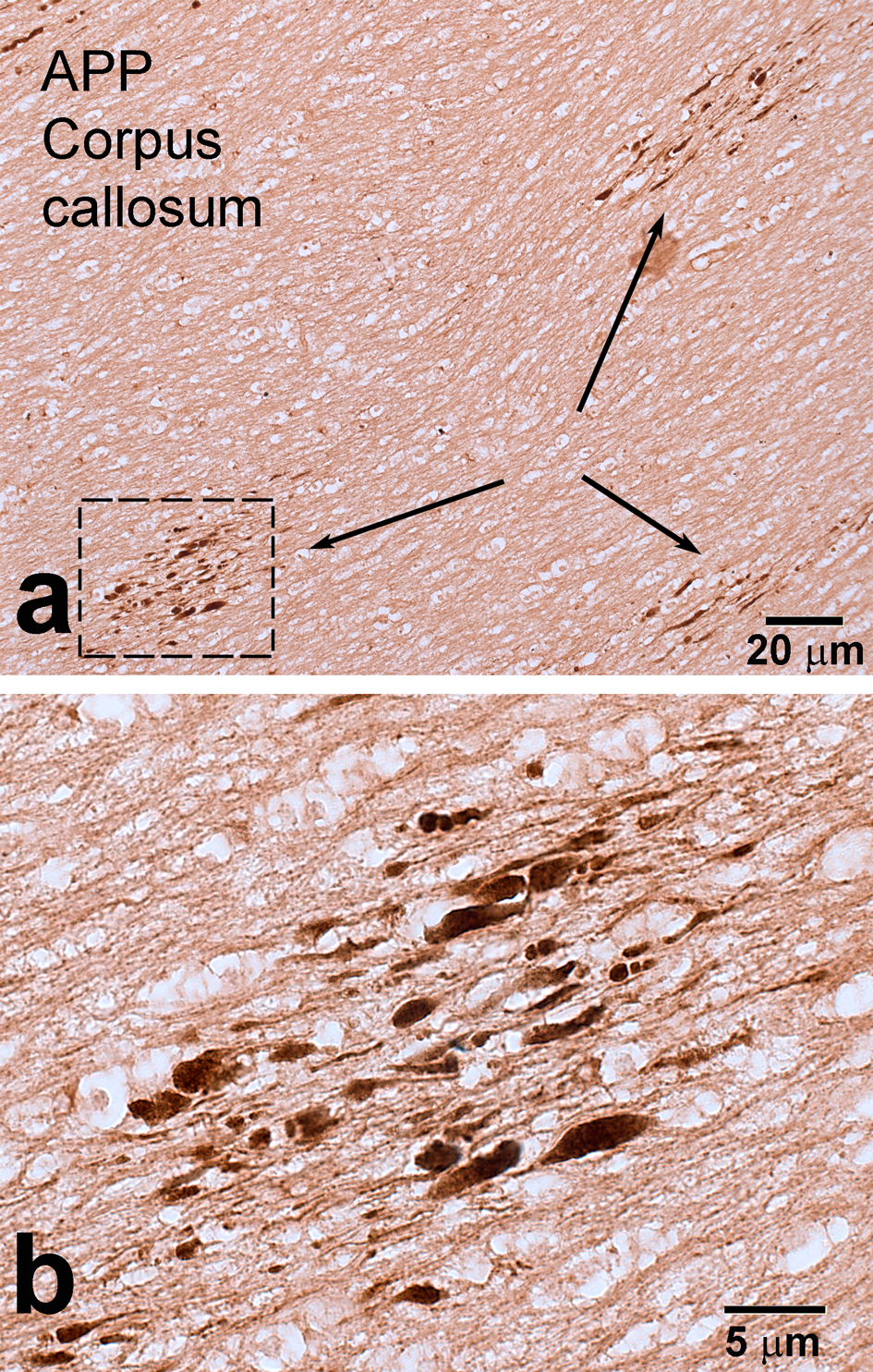

Supplement: Additional file 1: Figure S1. — APP (+) axonopathy in the corpus callosum of an index case with history of blast injury (Case 3, Table 1). Enlarged, fusiform axons and axonal bulbs are indicated with arrows in (a). (b) represents an enlargement of the blocked area in (a). It is unclear whether these patches of classical DAI are associated with blast or, rather, the concussion suffered as a result of assault 2 months prior to death. [file 40478_2014_153_MOESM1_ESM.tiff]

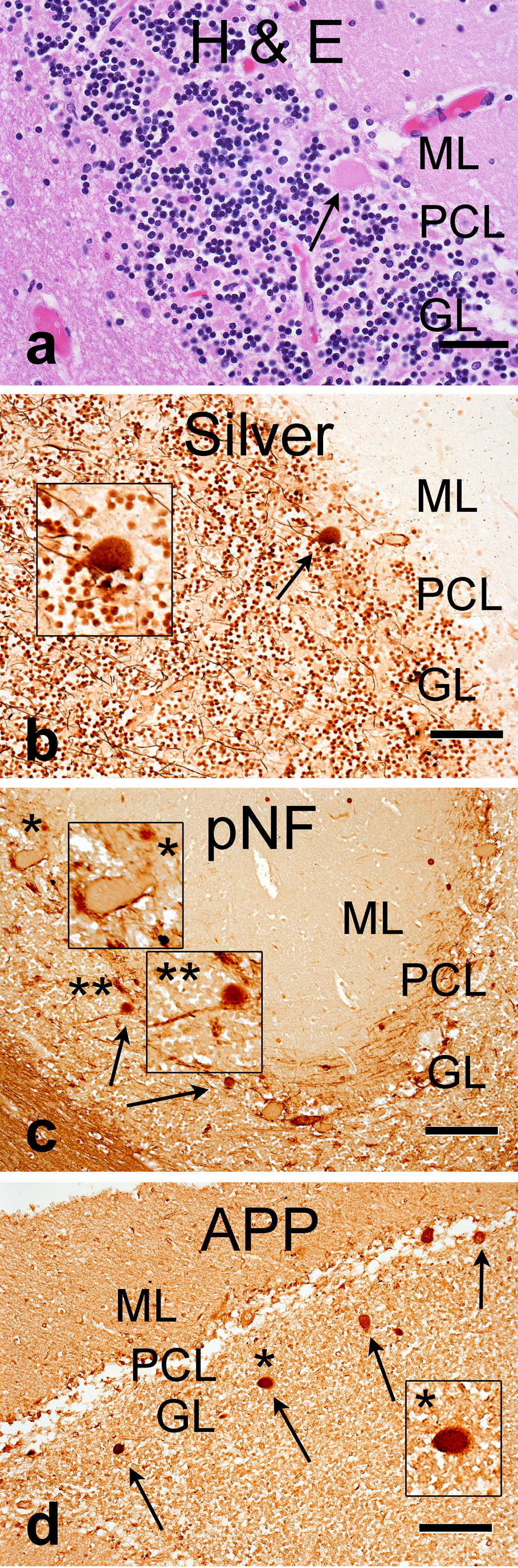

Supplement: Additional file 2: Figure S2. — Neuropathological findings in the cerebellum of an index case of blast injury (Case 3, Table 1) based on staining with hematoxylin-eosin (a), Gallyas silver (b), and phosphorylated neurofilament (c) or APP (d) immunohistochemistry. Note enlarged eosinophilic (a) or argyrophilic (b) or phosphorylated neurofilament- and APP-immunoreactive axon bulbs (c and d) in the Purkinje or granule cell layer next to the cell bodies of Purkinje cells (arrows). APP immunohistochemistry is especially sensitive in detecting these axonal abnormalities. Insets represent magnifications of areas indicated with asterisks. As in the case of classical axonal swellings with bulbs in Additional file 1: Figure S1, it is unclear whether these profiles are associated with blast or the concussion suffered from assault 2 months before death. GL, glomerular cell layer; ML, molecular layer; PCL, Purkinje cell layer. Size bars: 5 μm (a) and 20 μm (b, c and d). [file 40478_2014_153_MOESM2_ESM.tiff]

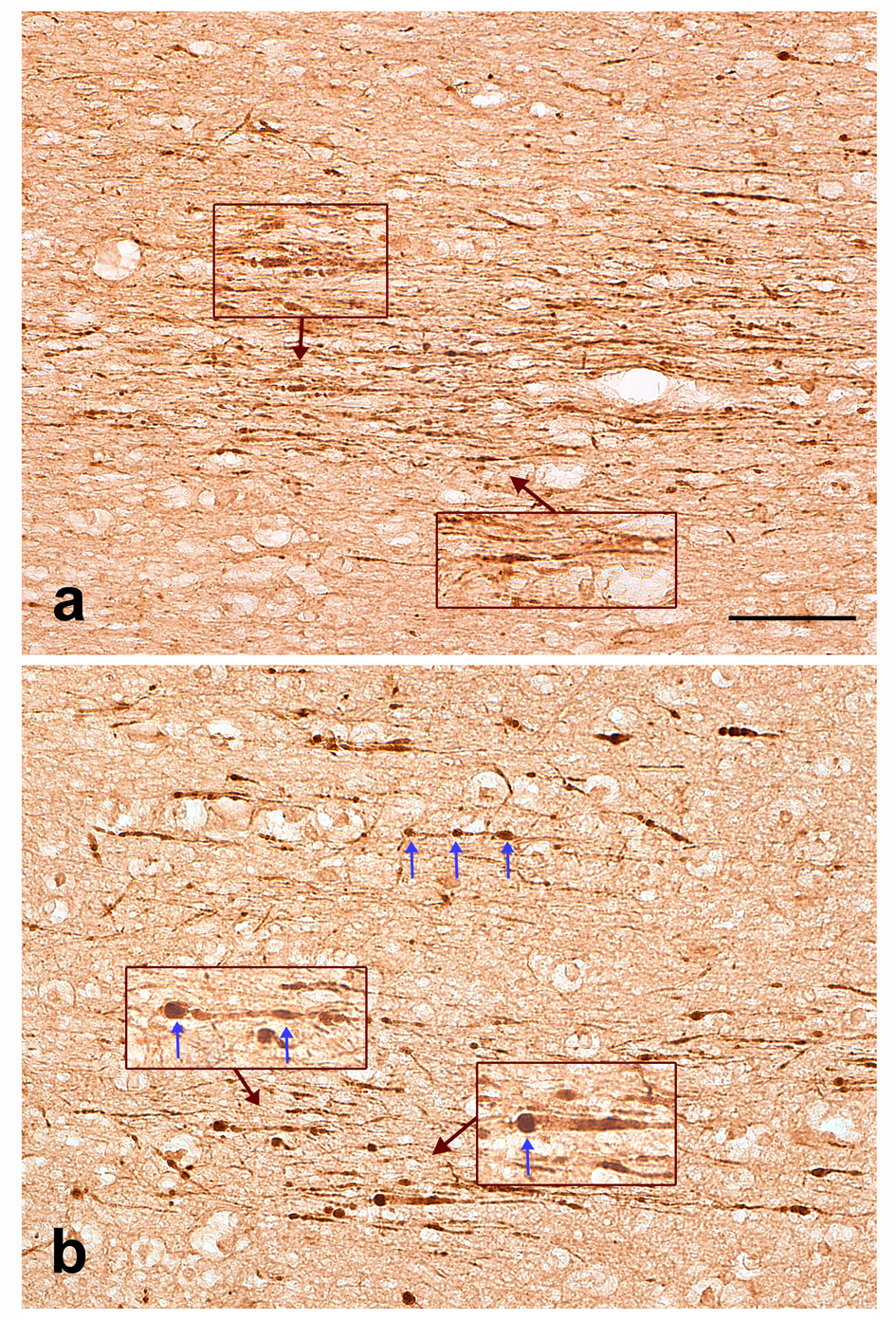

Supplement: Additional file 3: Figure S3. — This case of mixed drug overdose (Case 11 in Table 1) , including opiates, is associated with severe axonal injury that serves to illustrate better the type of axonopathy that occurs in drug intoxication. Although the pattern is qualitatively similar to that in opiate overdose, the intensity of labeling is unusually high. Note the straight thin axons with periodic round swellings (arrows in b). Size bars: 50 μm. [file 40478_2014_153_MOESM3_ESM.tiff]

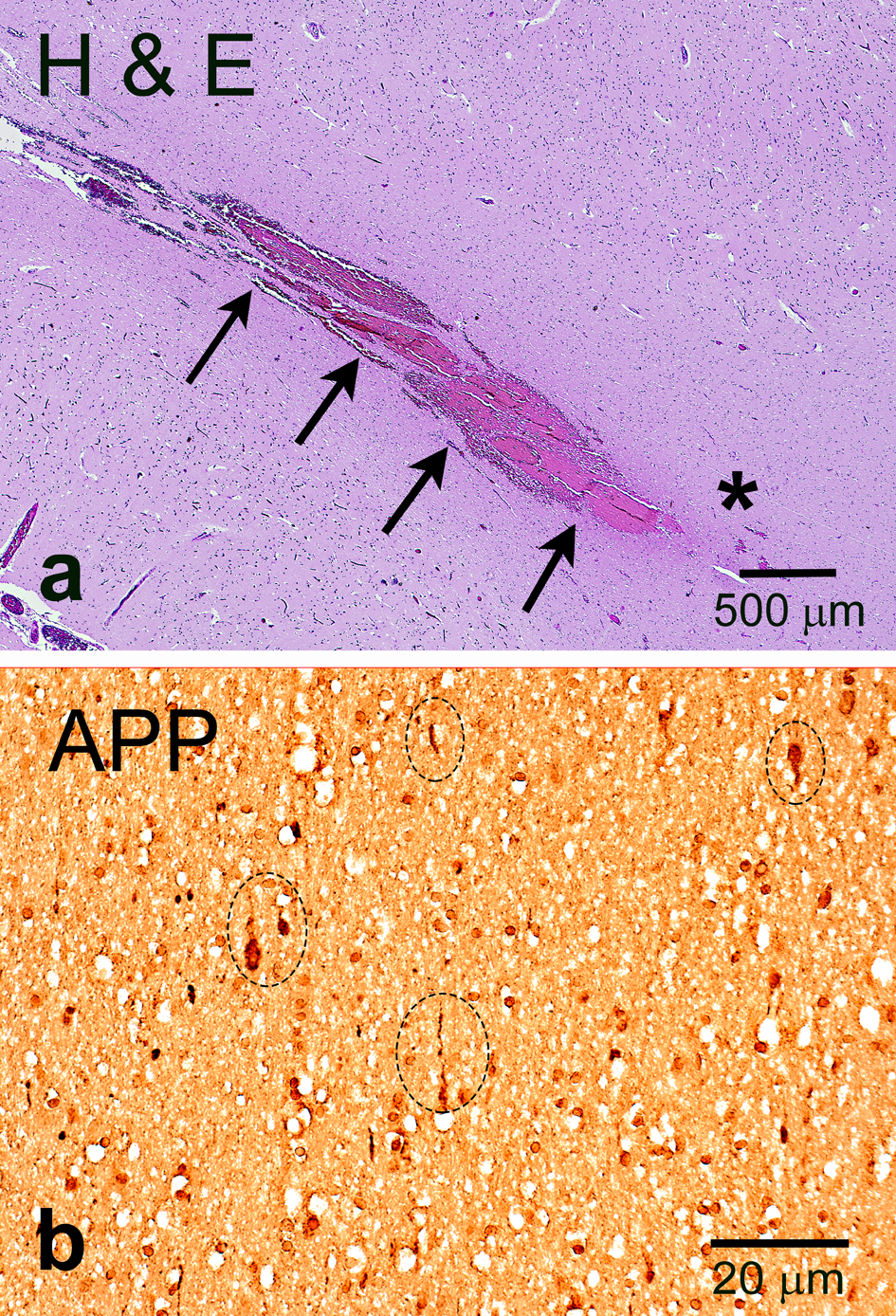

Supplement: Additional file 4: Figure S4. — Patterns of APP (+) axonal pathology in brain contusions. Case illustrated here (Case 19, Table 1) had hemorrhagic contusion in ventromedial frontal lobe (a, arrows). Note the presence of multiple APP (+) dilated, short axon stumps (circles) in the periphery of hemorrhage (area indicated with an asterisk in a). Size bars: 500 (a) and 20 (b) μm. [file 40478_2014_153_MOESM4_ESM.tiff]

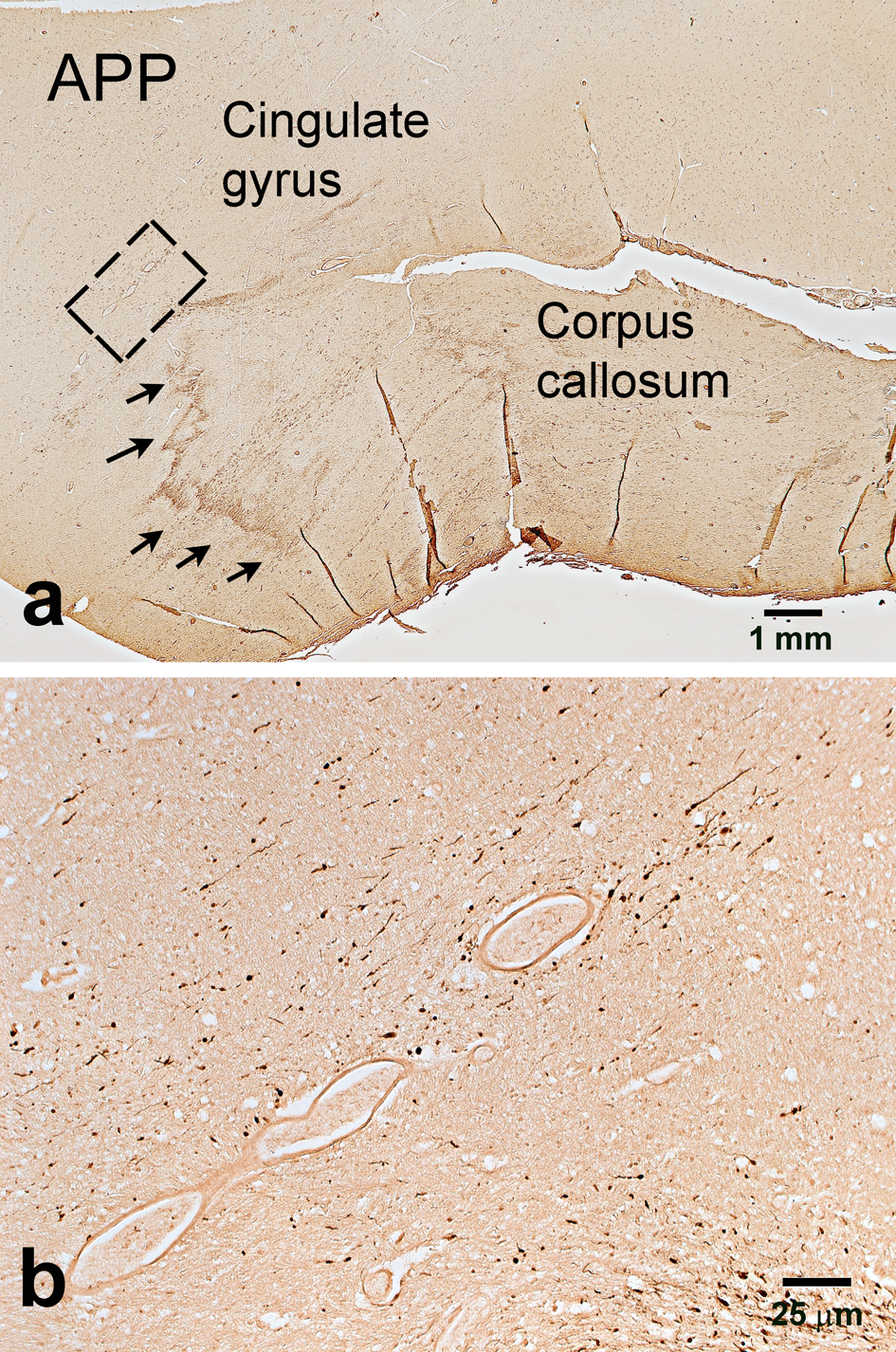

Supplement: Additional file 5: Figure S5. — APP (+) axonal pathology in cases of motor vehicle crashes is especially dense in medial dorsal frontal lobe (a, arrows). Images here correspond to case 20 (Table 1). Axonal abnormalities do not display perivascular patterns as in the case of blast injuries (b; this is an enlargement of framed area in a). Size bars: 1 mm (a) and 25 μm (b). [file 40478_2014_153_MOESM5_ESM.tiff]

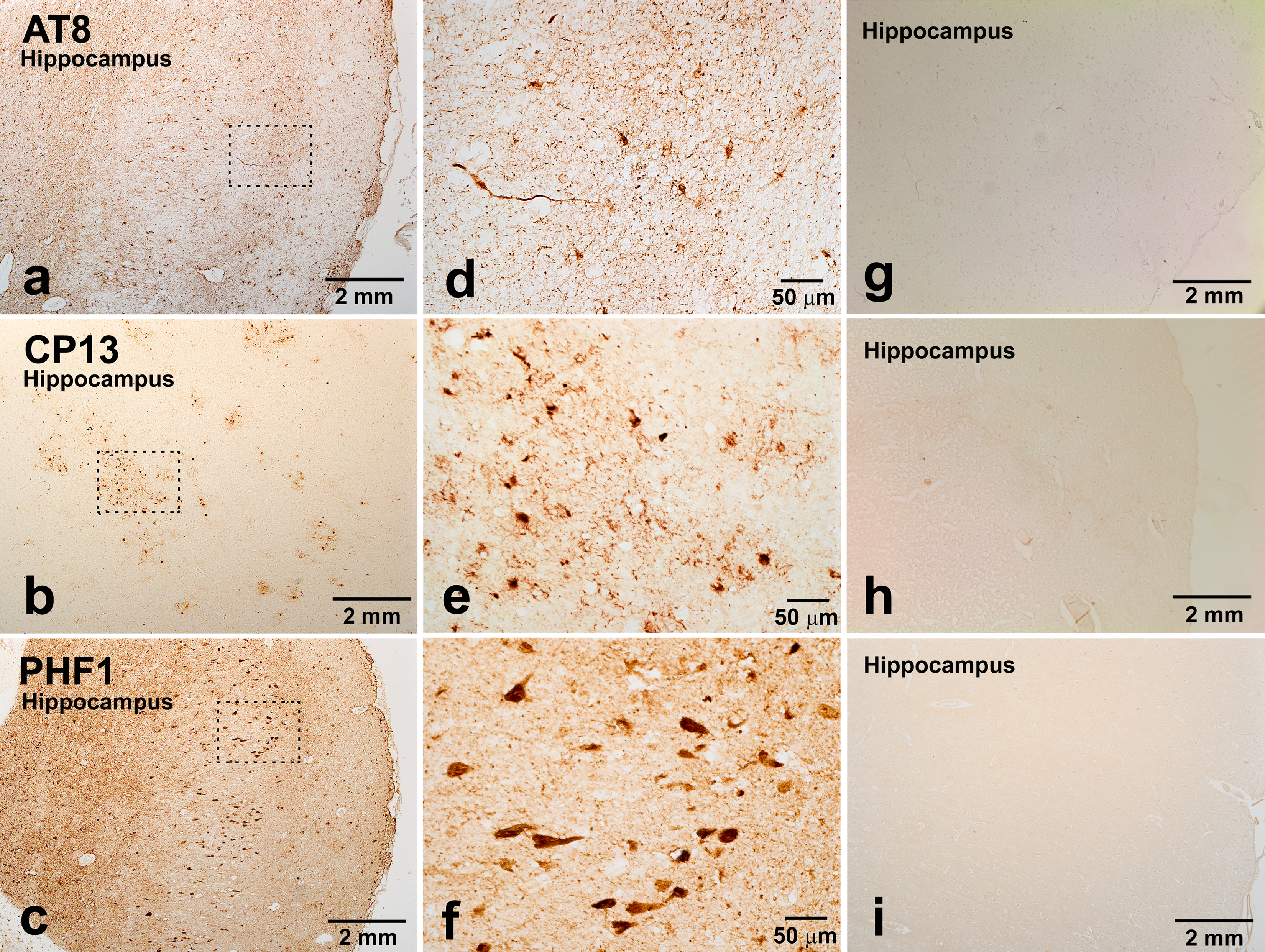

Supplement: Additional file 6: Figure S6. — Phosphorylated tau (AT8, CP13 and PHF1) staining of three blast injury cases (g, h and i) compared with an established dementia pugilistica case used here as positive control (a to f). Although antibodies AT8, CP13 and PHF1 react intensely with phosphorylated tau epitopes in the of the dementia pugilistica case (a-c; d-f are magnifications of framed areas in a-c, respectively), no immunoreactivity is seen in the hippocampus of Case 5 with history of blast exposure (g-i). Sections through the temporal lobe of other blast cases were similarly negative and MC1 antibody did not stain brain tissues with a history of dementia pugilistica or blast (data not shown). Size bars: 2 mm (a, b, c, g, h, and i) and 50 μm (d, e, and f). [file 40478_2014_153_MOESM6_ESM.tiff]
